# Designing local solutions for emptying pit latrines in low-income urban settlements (Malawi)

**DOI:** 10.1016/j.pce.2017.02.012

**Published:** 2017-08

**Authors:** W.C. Chipeta, R.H. Holm, J.F. Kamanula, W.E. Mtonga, F.L. de los Reyes

**Affiliations:** a Centre of Excellence in Water and Sanitation, Mzuzu University, Private Bag 201, Mzuzu 2, Malawi; b Department of Chemistry, Mzuzu University, Private Bag 201, Mzuzu 2, Malawi; c Department of Water Resources Management and Development, Mzuzu University, Private Bag 201, Mzuzu 2, Malawi; d Department of Civil, Construction, and Environmental Engineering, North Carolina State University, Campus Box 7908, Raleigh, NC 27695, USA

**Keywords:** Fecal sludge management, Gulper, Low-income countries, Pit emptying, Technology, Unplanned settlement areas

## Abstract

A lack of effective options in local technology poses challenges when onsite household sanitation facilities are eventually filled to capacity in unplanned settlement areas within Mzuzu City, located in northern Malawi. Vacuum trucks currently dominate the market but focus on emptying septic tanks in the more easily accessible planned settlement areas, rather than servicing the pit latrines common in unplanned settlement areas. As a result, households in the unplanned settlement areas within Mzuzu rely primarily on manual pit emptying (i.e., shoveling by hand) or digging a new pit latrine. These practices have associated health risks and are limited by space constraints. This research focused on filling the technological gap through the design, development, and testing of a pedal powered modified Gulper pump using locally available materials and fabrication. A modified pedal powered Gulper technology was developed and demonstrated to be capable of lifting fecal sludge from a depth of 1.5 m with a mean flow rate of 0.00058 m^3^/s. If the trash content was low, a typical pit latrine with a volume of 1–4 m^3^ could be emptied within 1–2 h. Based on the findings in our research Phase IV, the pedal powered Gulper modification is promising as a potential emptying technology for lined pit latrines in unplanned settlement areas. The success rate of the technology is about 17% (5 out 30 sampled lined pit latrines were successful) and reflects the difficulty in finding a single technology that can work well in all types of pit latrines with varying contents. We note that cost should not be the only design criteria and acknowledge the challenge of handling trash in pit latrines.

## Highlights

• Innovation in local pit latrine emptying technologies in Malawi is possible.
• Design criteria should include cost, safety, and effectiveness.
• Trash in pit latrines poses a challenge to emptying.
• The success rate of our technology is about 17%.

## 1 Introduction

In Malawi, pit latrines dominate in both urban and rural households for human waste disposal (National Statistical Office and ICF Macro, 2011). Removing fecal sludge (FS) from full pit latrines may be performed by either manual or mechanized techniques, which may include hand tools, vacuum trucks, pumping systems, or mechanical augers (Mikhael et al., 2014, Rogers et al., 2014, Thye et al., 2011). While the emptying method depends on the type of pit latrine, site accessibility, the type of equipment owned by the service provider, and the level of expertise, in many low-income countries, the top criteria is the local availability of the emptying method (Mikhael et al., 2014). This availability is also complicated by the fact that physical properties are variable between and within pit latrines (Radford and Fenner, 2013).

In Malawi, national and local legislation covering the removal of FS from onsite household sanitation facilities is weak (Holm et al., 2015). Our research focused on Mzuzu City, located in northern Malawi. The city population is estimated to have reached 157,612 people in 2015 based on annual growth trends (UN-Habitat, 2011). Mzuzu City has no sewage system. Rapid urbanization has led to the formation of several low-income unplanned settlement areas within the city limits, mostly on the periphery of the city. Within the city, 48% of the population lives in informal settlements, and 94% of residents in these areas use pit latrines or septic tanks (Mzuzu City Council, 2013). The Mzuzu City Council is unable to provide adequate fecal sludge management (FSM) services due to limited financial resources, technology options, and technical capacity (UN-Habitat, 2011). Therefore, sanitation entrepreneurs using vacuum trucks address this need, primarily focusing on emptying septic tanks in the easily accessible formal areas of the city. Other globally available technology options, such as the power earth auger and manual diaphragm pumps, are not available to sanitation entrepreneurs in Mzuzu. Hence, households in the informal urban settlements within Mzuzu primarily rely on the current locally available options, predominantly manual pit emptying (i.e., shoveling by hand and illegal disposal) or digging a new pit latrine. These practices have associated health risks and are limited by space constraints.

The lack of effective and locally available emptying technologies hinders efforts toward improving sanitation and public health in unplanned settlement areas within Mzuzu City, and is likely similar to the situations observed in other low-income countries. This research focused on filling the technology gap through innovation involving the design, development, and testing of a pedal powered Gulper modification using locally available materials and fabrication.

## 2 Materials and methods

### 2.1 Research design

The design, development, and field testing of a novel pedal powered Gulper technology on actual pit latrines in Mzuzu was undertaken from February 2014 to June 2015. A trial and error process was used, and quantitative data on the performance of the pedal powered Gulper pump was collected at each phase. Research observations and lessons learned were obtained and continuously assessed.

### 2.2 Study location

Area 1B is a high-density low-income informal residential urban settlement within Luwinga ward on the northern edge of Mzuzu City, Malawi. The study settlement has a population of 319 households (Mzuzu City Council, 2013).

### 2.3 Sampling method

To test the modified Gulper technology in Area 1B, purposive sampling was used to select 30 lined household pit latrines. The method was chosen because sludge characteristics in pit latrines vary, regardless of being from the same city, area or even adjacent households (Niwagamba et al., 2014). Additionally, differences in user practices, such as diet and anal cleansing material, as observed by Still and Foxon (2012) in South Africa, similarly apply to the sampled pit latrines within Area 1B (Chiposa, 2016). This study attempted to limit this variability by using a geographically limited study area. Because unlined pits have the potential to collapse during pit emptying, they were excluded from the study.

### 2.4 Materials

We attempted to improve on two existing technologies available in Malawi: the treadle pump used throughout Malawi for irrigation, and the arm powered Sludge Gulper developed by the London School of Hygiene and Tropical Medicine and designed for pit emptying (Mikhael et al., 2014). Limitations of the Gulper included its length (1.5 m), decreased effectiveness with denser sludge, and slow rate of emptying. Current ongoing modifications of the Gulper by Water for People (2014) and the Rammer technology were excluded in this study. This research focused on development of local innovation to pump FS.

### 2.5 Design process of pit emptying technology

Context-specific design criteria were formulated to develop an effective household pit emptying technology for low-income informal urban settlements in Mzuzu:

• Materials and spare parts of the technology developed must be locally available
• Extraction time of FS should be within 1–2 h
• Health risks for operator and serviced household should be lower than those for manual pit emptying
• Portable (less than 50 kg)
• Able to pump FS from a depth of 1.5 m
• Effectively remove trash in the pit latrines
• Achieve a discharge rate of 0.001 m^3^/s
• Simple to operate (requiring no formal education)
• Cost of technology should be less than U.S. $200

A conceptual design was developed based on the criteria, and the required materials were obtained for fabrication by local welders. The fabricated technology was first tested on a mud slurry, used to simulate FS, to determine initial performance parameters. These parameters facilitated the evaluation of each phase against the established design criteria. Based on the evaluation assessments, the concept was subsequently improved and redesigned in each successive phase. Only Phase IV was tested on household pit latrines.

### 2.6 Testing procedure

The procedure for the pit latrine testing in Area 1B was as follows:

*Step 1:* Briefing the testing team on health and safety issues
*Step 2:* Administering the consent form for each household owner/occupant of the sampled latrines
*Step 3:* Inspecting latrine structure for cracks
*Step 4:* Measuring the latrine superstructure (door, squat/key hole, floor slab)
*Step 5:* Removing trash with a manual hook. Two types of manual hooks were used, one shaped as a claw (three U-shaped hooks) and the other a sweeping brush with 6-inch nails. Both had a maximum height of 2 m and a 40-cm handle. The volume of trash removed per pit was measured using a 20-L (0.02 m^3^) pail.
*Step 6:* Fluidizing sludge in the pit latrines using water. Fluidization was performed in increments of 0.02 m^3^ by volume using manual agitation with a trash removal hook to improve consistency.
*Step 7:* Measuring flow rates of the modified pedal powered Gulper by filling a 20-L pail until the maximum Gulper length of 1.5 m was reached.
*Step 8:* Cleaning the test site around the pit latrine
*Step 9:* Disposing FS at city sludge ponds
*Step 10:* Cleaning and sanitizing pit emptying equipment

### 2.7 Analysis

Statistical analysis was performed using Excel 2013 and Statistical Package for Social Sciences (SPSS) version 16.0.

### 2.8 Ethics

The study received ethical clearance from the Republic of Malawi National Commission for Science and Technology (NCST) (Protocol P.10/14/22).

## 3 Results

### 3.1 Design and development phases of pit emptying technology

**Fig. 1:**
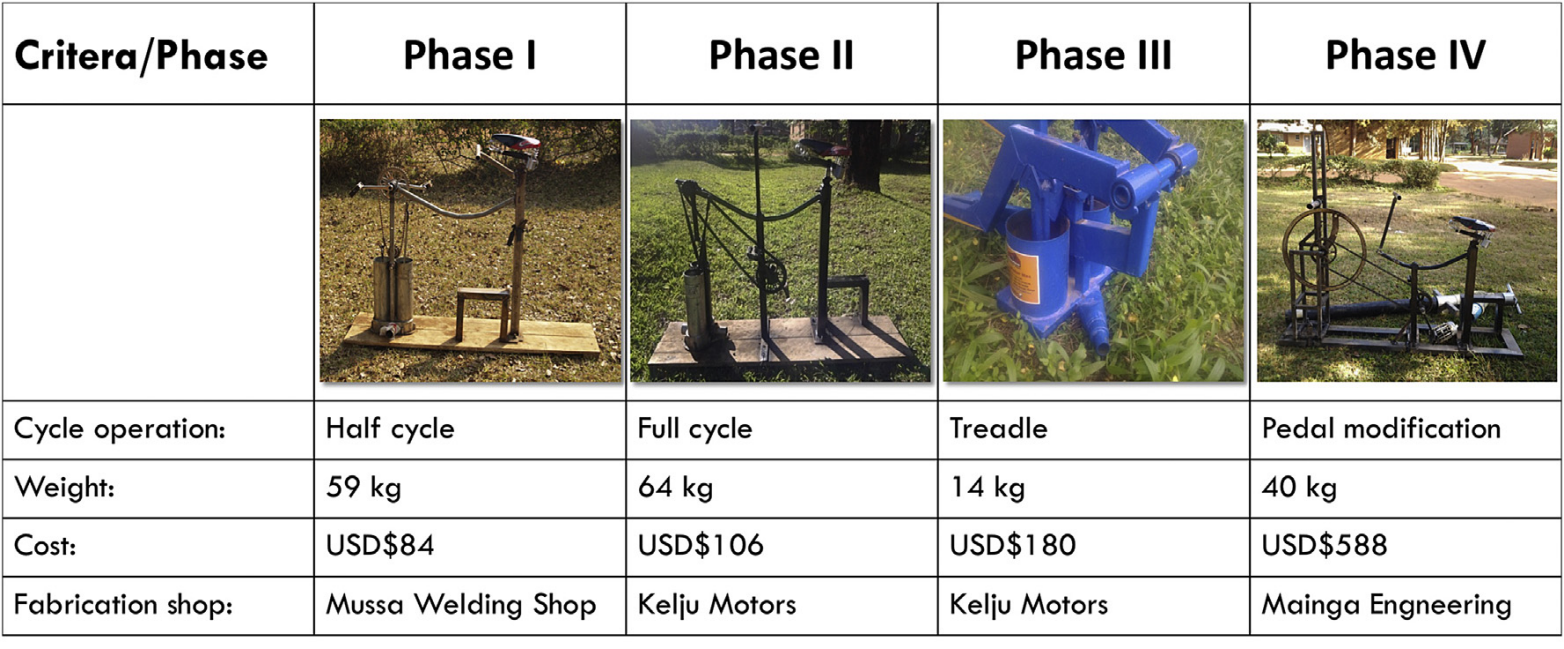
Summary of design phases and criteria.

Fig. 1 shows the three phases assessed for treadle pump modification. The fourth phase integrated lessons learned in the previous phases.

### 3.2 Design schematic diagram of Phase IV

**Fig. 2:**
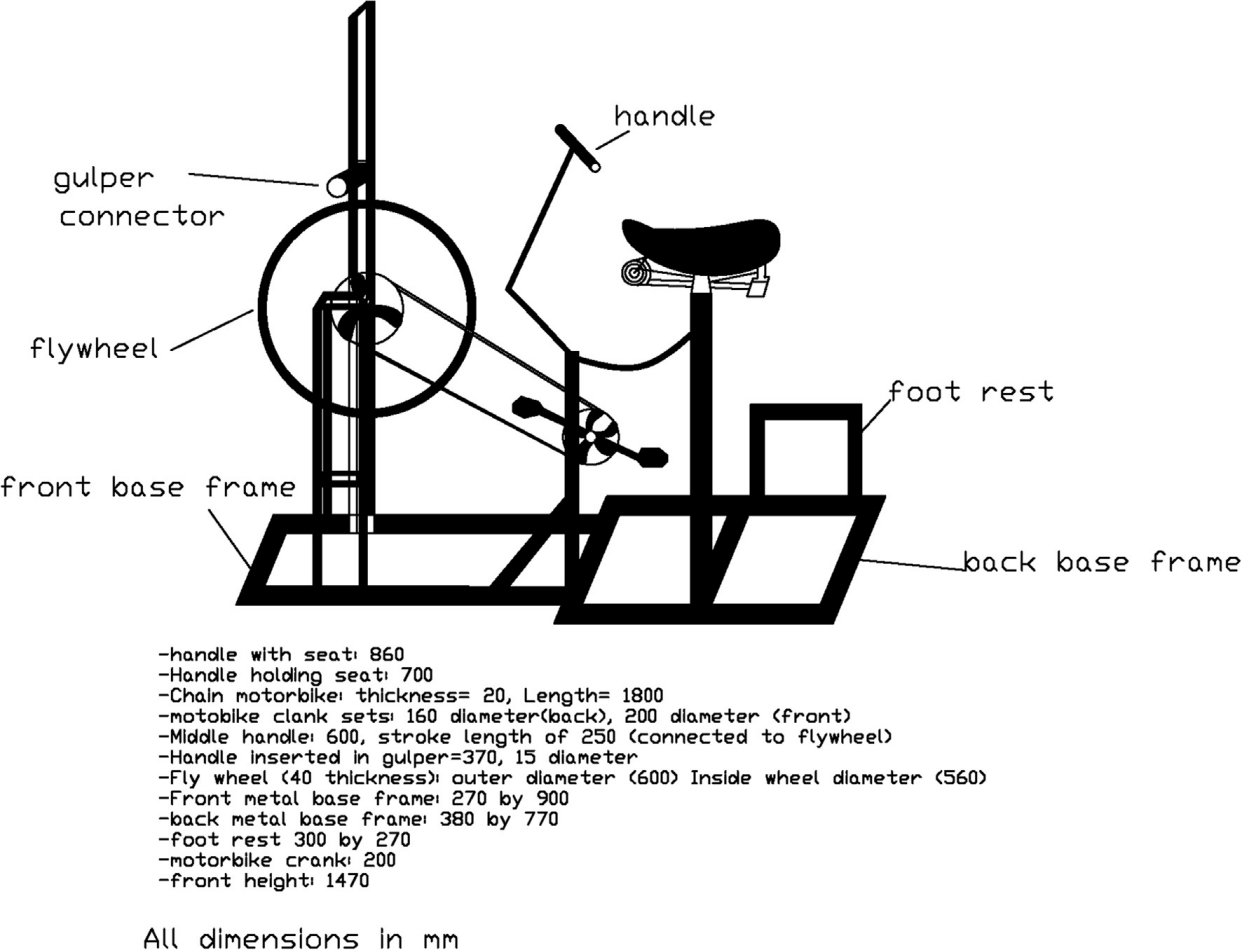
Schematic of Phase IV pedal powered Gulper modification.

Fig. 2 shows the Phase IV design which explored pedal powered Gulper modification, building on the full cycle operation tested in Phase II with the addition of a flywheel.

### 3.3 Testing of pit emptying technology at Area 1B

Table 1 highlights the characteristics of the five pit latrines that were successfully emptied using our design with a maximum Gulper length of 1.5 m and a riser pipe of 100 mm.

**Table 1:** Dimensions of five successfully emptied pit latrines.^a^

Pit#	Pit slab (cm)	Squat/key hole (cm)	Door dimensions (cm)
1	91 × 175	32 × 16	61 × 164
2	115 × 92	18 × 16	60.5 × 171
3	125 × 90	29 diameter (round)	52 × 150
4	136 × 110	20 × 20	67 × 175
5	166 × 145	16 × 20	Temporary superstructure

a The remaining 25 pit latrines in the study were not successfully emptied.

**Fig. 3:**
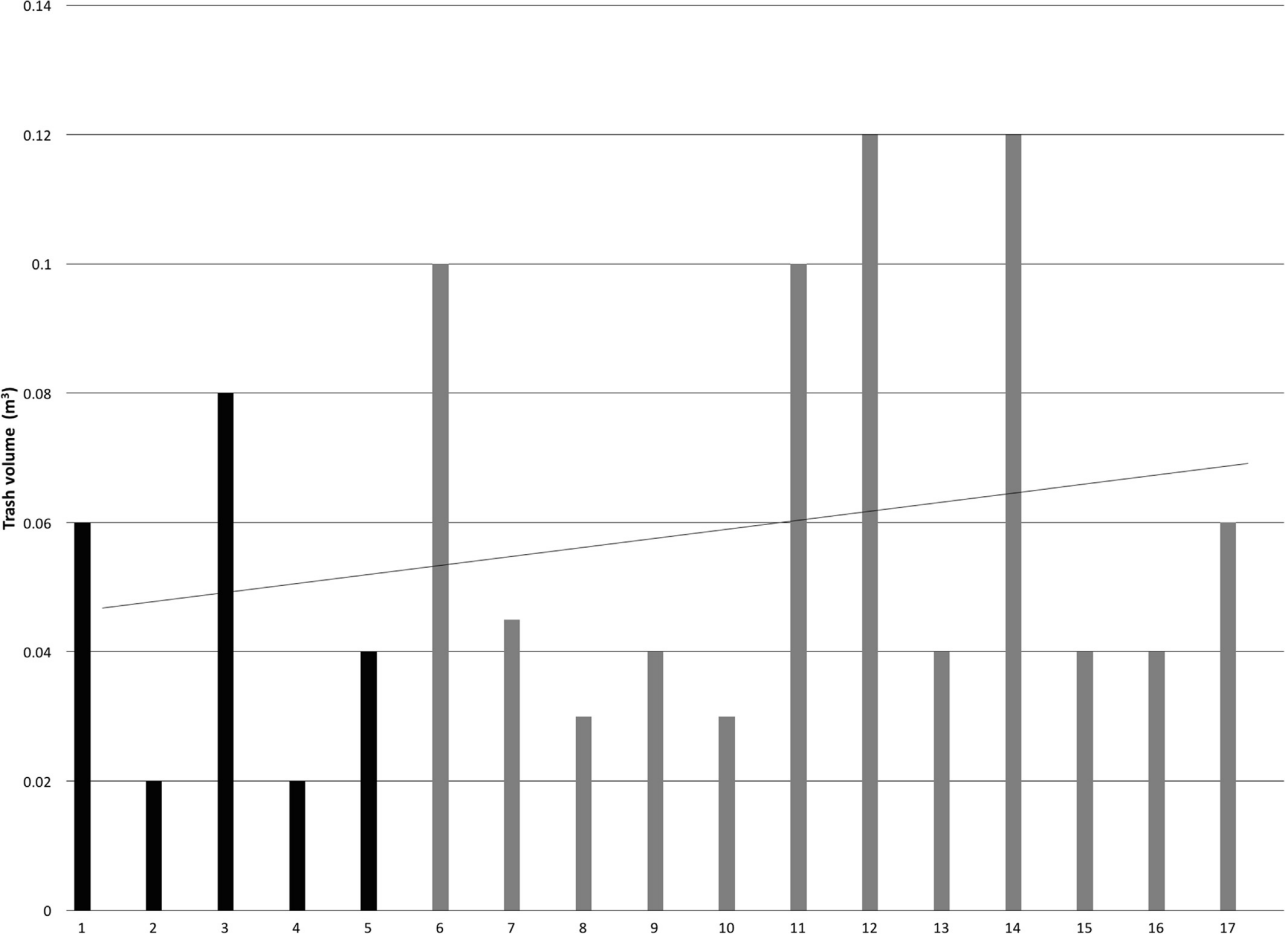
Volume of trash fished from the pit latrines (five pit latrines successfully emptied highlighted in black).

The volume of trash ‘fished’ from the pits (*n* = 17) ranged from 0.02 to 0.12 m^3^ with a mean of 0.058 m^3^ and a standard deviation of 0.03 (Fig. 3).

Table 2 depicts the mean time by task for the pit emptying operation using the pedal powered Gulper technology. The pumping time of 12 ± 3 min was the quickest portion of all tasks performed, while the trash removal task with a mean time of 21 ± 11 min took the longest.

**Table 2:** Time by task of pit emptying operation for five successfully emptied pit latrines.

Task	Minutes						
	Pit 1	Pit 2	Pit 3	Pit 4	Pit 5	**Mean**	**Std dev**
Assembly in field	15	10	20	15	20	**16**	**4**
Fishing trash	20	10	40	20	15	**21**	**11**
Fluidization	10	10	40	10	10	**16**	**13**
Sludge pumping	10	15	10	10	15	**12**	**3**
Unclogging pump	15	10	45	0	10	**16**	**17**
Disassembly	20	10	25	15	15	**17**	**5**
Cleanup in field	15	15	20	20	15	**17**	**2**
Total Time	105	80	200	90	100	**115**	**48**

**Fig. 4:**
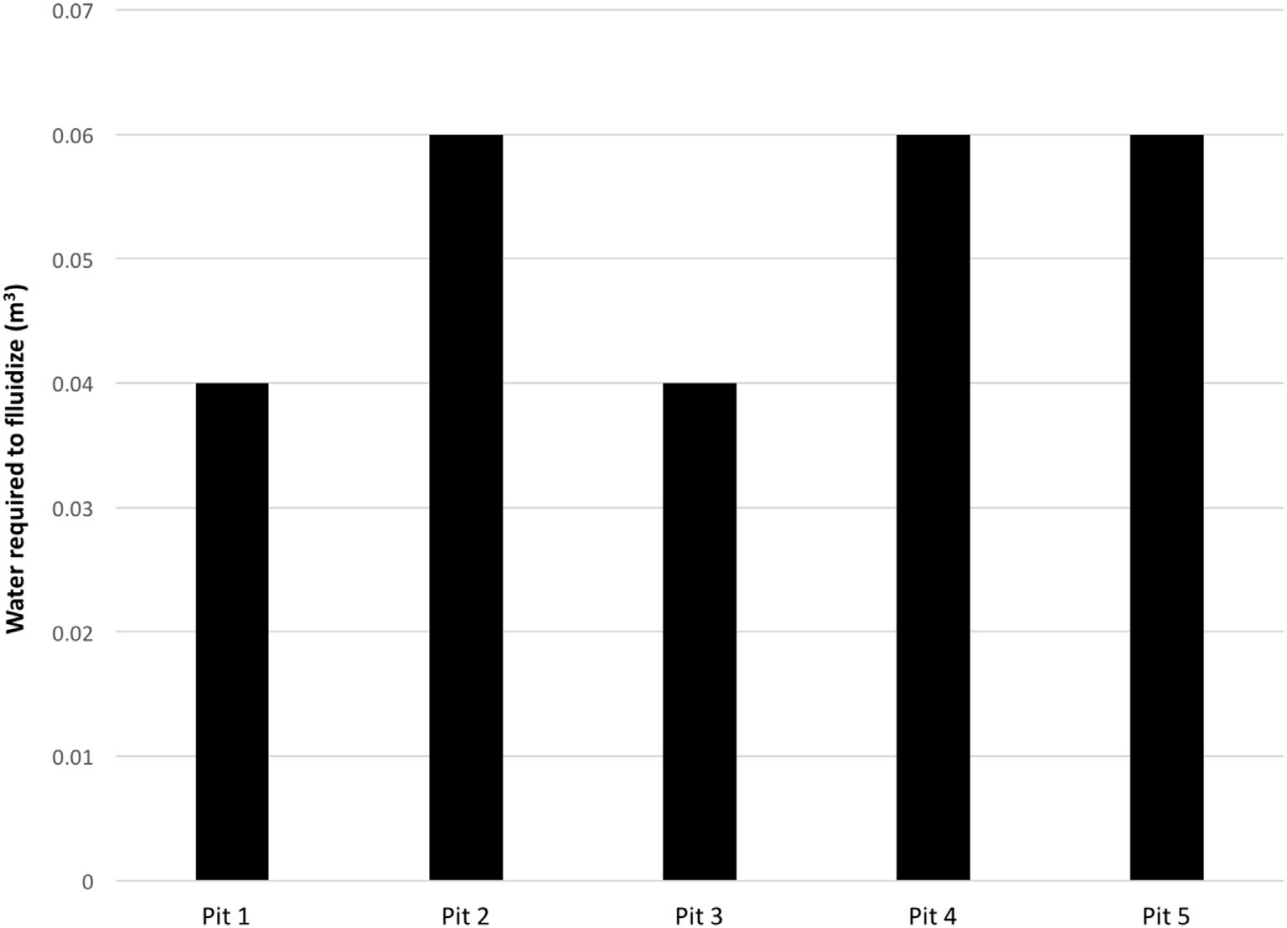
Volume of water used for fluidization in pit emptying operation.

Fig. 4 shows that the volume of water used for the fluidization prior to sludge pumping ranged from 0.04 to 0.06 m^3^. The mean volume of water used for the fluidization of FS was 0.052 ± 0.010 m^3^.

**Fig. 5:**
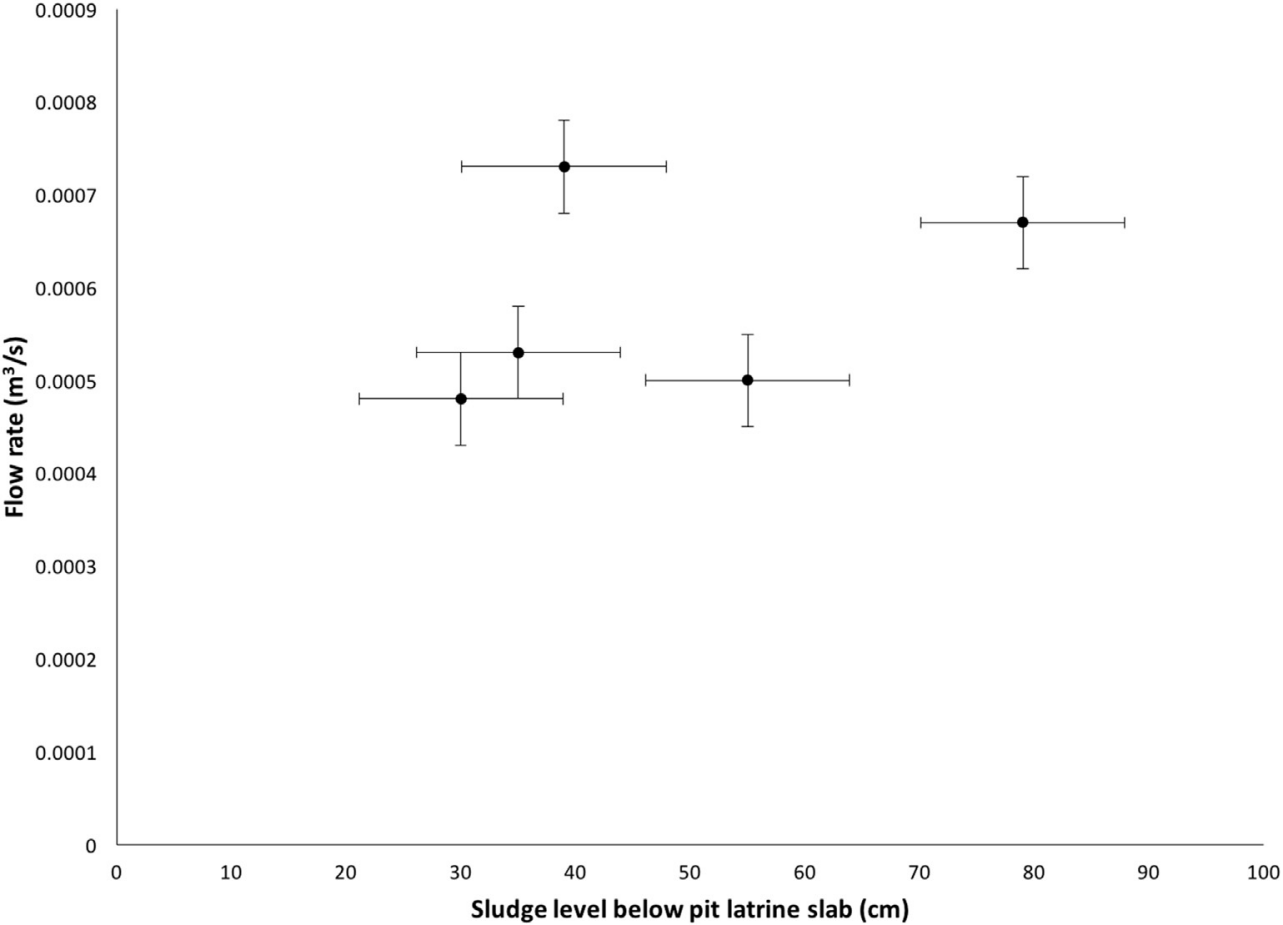
Flow rates of pit emptying operation (error bars represent standard deviation).

Fig. 5 shows that the mean flow rate of the pedal powered Gulper design was 0.00058 m^3^/s with a standard deviation of 0.00013. The differences in mean flow rates were not significant (p value of 0.05).

## 4 Discussion

The purpose of our trials was to come up with a design that will work for a large set of pit latrines. Thus, research trials of technology under real conditions decrease the cost for users and operators through attempts for further improvement in the design of the technology.

### 4.1 Phase I

The first phase design involved the use of a bicycle chain (1 cm thickness) to drive the pulley. A chain was selected due to its durability and reliability compared to a rope. Bicycle pedals replaced the wooden foot treadles to enable a continuous cycle of motion. A bicycle crank set was added to enable rotation at a half cycle motion upon the application of force to the pedals. A bicycle hub was attached at the crank to transfer the pedal motion to the chain. Lubricant oil (engine oil #40) was used to ease the motion of the flap handle in the cylinders. A bicycle seat and handles were added to enable foot operation of the pump over longer periods of time.

The machine was locally fabricated by Mussa welders (a small-scale roadside welder) at a cost of U.S. $84, which was well within the design criteria of being under U.S. $200.

Pumping tests on the mud slurry using the Phase I modified pump indicated that the pump was unable to lift a mud slurry from a 1 m hand dug pit and provided negligible flow rates. Instead, the mud was deposited in the lining of the suction hose and clogged the valves. The inability to maintain a vacuum limited suction, resulting in low flow rates, deposition in the suction hose, and subsequent clogging. In addition, the bicycle chain broke several times during the trials.

The treadle pump technology operated on a vacuum based system, which required priming with water to enable the suction. It proved difficult to maintain a vacuum, as leakages in the system caused a frequent loss of suction. The half cycle leg motion made operation tedious and ineffective. The weight of the Phase I design was 59 kg, above the 50-kg weight established in the design criteria to enable easy manual transportation to the work site.

Based on the above observations, the research team carried out a redesign to enable full cycle crank motion.

### 4.2 Phase II

The fabrication shop used in Phase I struggled with a lack of engineering expertise to implement the envisioned full cycle motion design modification. Thus, Phase II of the treadle pump was fabricated by Kelju Motors. In contrast to small-scale roadside welders, Kelju Motors focused on motor vehicle repairs. These welders approached the task as a challenge, displaying a positive attitude during innovation development. However, the shop was still limited by a lack of equipment, such as a grinder, requiring renting equipment from other nearby welders. This resulted in schedule delays. Despite these delays, Kelju Motors was able to eventually incorporate a full cycle pedaling motion, similar to that of riding a bicycle.

Modifications undertaken included the replacement of the bicycle chain with a motorcycle chain. Tests on the mud slurry were conducted, but again, leakage of the mud slurry within the system prevented the accurate determination of flow rates.

The full cycle pedal motion with leg power proved to be easier to operate than the half cycle motion in the Phase I design. However, due to the increased load, the mud pumped out during pedaling posed a challenge.

The weight of the Phase II machine was 64 kg, due to the addition of parts, such as the motorbike chain.

Based on the above results, the research team undertook a redesign to (1) add a flywheel to assist with pumping power and (2) reassess the conventional treadle operation from water to sludge.

### 4.3 Phase III

Phase III explored conventional treadle pump operation using a Super Money Maker treadle pump. The new machine was also fabricated by Kelju Motors. However, the workmanship of the implemented design changes was of substandard quality, with major leakage from the base container. This leakage was unacceptable because of the health risks via FS exposure for the operator and the serviced household. The challenges observed in this phase were similar to those in Phase I and II. The vacuum based technology of the treadle pump and valves were designed for pumping water, rather than denser fluids, and the tests on the mud slurry failed.

Based on the above observations, the research team reviewed the three phases of the treadle pump modification. Phase II, with the full cycle motion, showed the most potential to reduce the effort exerted during operation, especially if a flywheel could be added. Hence, the full cycle option was moved forward to Phase IV.

### 4.4 Phase IV

Phase IV explored the pedal powered Gulper modification with full cycle operation and a flywheel. In this phase, the design was fabricated using the most capable engineering fabricators in Mzuzu, Mainga Engineering. Mainga Engineering is located in an industrial area of Mzuzu City near the local tobacco trading floor, the Coca-Cola bottling company, and local coffee distributors. Notably, the shop had onsite equipment including grinders as well as engineering expertise. The full cycle motion was successfully implemented, with the addition of the flywheel, enabling smooth leg operation of the pedal powered Gulper pump.

The cost of materials and fabrication was U.S. $588, which was above the U.S. $200 limit established in the design criteria and may still render the technology not affordable especially for the Malawian population (unplanned settlement areas) for which this technology is being targeted. Mainga Engineering's higher capacity workshop with qualified engineers involved higher labor charges than the previous fabricators. It should be noted that the cost of a prototype is always higher, and once more machines are manufactured, the cost is expected to decrease. The cost recovery is based on a business model where the payments for pit emptying service can pay for the machine over a specified number of years. A detailed business model with financing options, interest rates, and other factors, is needed to test the financial feasibility for pit emptiers. We note that Gulpers are being used by pit emptying teams in many African countries (Water for People, 2014), and the additional cost in our machine is primarily linked to the bike components and frame.

However, the focus on lined pit latrines in our design is limiting because most pit latrines in Malawi are not lined. Alternative arrangements, such as supports where the machine can rest and the machine is not weighing down directly on the floor, need to be further explored.

### 4.5 Testing of pit latrine emptying technology

During testing of the modified pedal powered Gulper technology, access to the sampled pit latrines in the study area (Table 1) was through the squat/key hole in the floor of the pit latrine. In one of 30 cases, the latrine had a blind wall in the front, which prevented access for emptying.

Furthermore, trash was found in all of the sampled pit latrines (Fig. 3) with a mean volume of 0.06 m^3^. The trash included stones, rags, sanitary pads, condoms, bricks and glass. A similar concern regarding the role of trash in the filling rate of pit latrines was also documented in South Africa by Brouckaert et al. (2013). In our study, after removing the trash, water was added (Fig. 4) to fluidize the FS to improve the pumping flow rate. Yet, trash still clogged the inlet of the Phase IV modified pedal powered Gulper technology. Unclogging by hand was required due to the absence of a reversing mechanism, increasing the pit emptying operation time (Table 2) and causing the emptying process to be messy.

The flow rates for the pedal powered Gulper were below the established design criteria of 0.001 m^3^ (Fig. 5) with a mean flow rate of 0.00058 m^3^/s. However, this flow rate was slightly above the conventional operation of the arm powered Sludge Gulper of 0.0005 m^3^/s, as reported by Mikhael et al. (2014). Our technology was also within the range of the performance of the Manual Pit Emptying Technology (MAPET), which operates at 0.00017 m^3^/s to 0.00067 m^3^/s (Mikhael et al., 2014).

Our design requires further review to optimize the power transmission mechanism to be able to attain higher pumping rates. Another limitation is the localized nature of the available materials and labor when compared to other countries (Thye et al., 2011) where similar manual or machine technologies have been used.

## 5 Conclusions

The success rate of our technology is about 17% (5 out 30 sampled lined pit latrines were successfully emptied) and reflects the difficulty in finding a single technology that can work well in all types of pit latrines with varying contents. Although manually powered machines will always have low success rates because of trash, sludge thickness, and sludge variability, that should not stop the development of such machines. However, not all local fabricators are interested in developing technologies.

Through a four-phase technology development process, a modified pedal powered Gulper technology was developed from local materials and used to remove FS from pit latrines in unplanned settlement areas within Mzuzu. The technology could lift FS from a 1.5 m depth with flow rates of 0.00058 m^3^/s. A typical pit latrine volume of 1–4 m^3^ could be emptied within 1–2 h. This speed is attainable only if the trash content is low. Health risks for the operator and serviced household were reduced compared to manual pit emptying, and the system was simple to operate (requiring no formal education).

Based on the results of Phase IV, the pedal powered Gulper modification can be a promising technology that may be further refined to increase success rates, lower the cost of materials, and optimize the operation. The following are recommended for further research:

• Review the developed pedal powered Gulper technology design to maximize ergonomics, yielding higher pumping rates and optimizing power transfer
• Modify the design to enable pumping outside the latrine with the pedal powered mechanism
• Accommodate pit latrines with small doors with a narrower equipment design
• Add an extended discharge hose, 1–2 m to the length, enabling direct emptying into large containers which may allow continuous pumping operations
• Assess availability of high capacity fabricators in other areas of Malawi
• Investigate social and technical solutions to prevent trash from being placed in pit latrines to enable the pedal powered Gulper modification and other pit emptying technologies to pump FS from pit latrines more effectively in Mzuzu.

When the highest quality fabricators are used, designing local solutions for pit emptying in low-income urban settlements of countries such as Malawi is possible. We note that cost should not be the only design criteria and acknowledge the challenge of handling the trash in pit latrines. While the technology was perhaps not as optimized as other globally available pit emptying options, this study has demonstrated that local innovation under real conditions is part of the global sanitation solution.

## Funding

This study was supported by Research Project KSA11:K5/2296/11, which was awarded to the Mzuzu University Centre of Excellence in Water and Sanitation and focused on “Solutions for Pit Desludging and Subsequent Sludge Management in Low Income Urban Settlements in Malawi” with support from the Water Research Commission of South Africa.
